# Chitosan Hydrogels Based on the Diels–Alder Click Reaction: Rheological and Kinetic Study

**DOI:** 10.3390/polym14061202

**Published:** 2022-03-16

**Authors:** Cinthya Ruiz-Pardo, Luisa Silva-Gutiérrez, Jaime Lizardi-Mendoza, Yolanda López-Franco, Carlos Peniche-Covas, Waldo Argüelles-Monal

**Affiliations:** 1 Grupo de Investigación en Biopolímeros, Centro de Investigación en Alimentación y Desarrollo A.C., Hermosillo 83304, Sonora, Mexico; cinthya.ruiz@estudiantes.ciad.mx (C.R.-P.); lsilva@ciad.mx (L.S.-G.); jalim@ciad.mx (J.L.-M.); lopezf@ciad.mx (Y.L.-F.); 2 Facultad de Química, Universidad de la Habana, Vedado, La Habana 10400, Cuba; cpeniche2015@yahoo.com

**Keywords:** click chemistry, Diels–Alder reaction, chitosan, PEG-maleimide, furan, rheology

## Abstract

The Diels–Alder reaction is recognized to generate highly selective and regiospecific cycloadducts. In this study, we carried out a rheological and kinetic study of *N*-furfuryl chitosan hydrogels based on the Diels–Alder click reaction with different poly(ethylene)glycol-maleimide derivatives in dilute aqueous acidic solutions. It was possible to prepare clear and transparent hydrogels with excellent mechanical properties. Applying the Winter and Chambon criterion the gel times were estimated at different temperatures, and the activation energy was calculated. The higher the temperature of gelation, the higher the reaction rate. The crosslinking density and the elastic properties seem to be controlled by the diffusion of the polymer segments, rather than by the kinetics of the reaction. An increase in the concentration of any of the two functional groups is accompanied by a higher crosslinking density regardless maleimide:furan molar ratio. The hydrogel showed an improvement in their mechanical properties as the temperature increases up to 70 °C. Above that, there is a drop in G’ values indicating that there is a process opposing to the Diels–Alder reaction, most likely the retro-Diels–Alder.

## 1. Introduction

Chitosan is one of the most abundant polysaccharides in nature. It exhibits biological properties such as low toxicity, mucoadhesivity, biocompatibility, and biodegradability, which make it attractive for use as a drug delivery vehicle [1,2]. Due to the presence of abundant amino (–NH_2_) and hydroxyl (–OH) groups, chitosan can be chemically crosslinked giving rise to hydrogels with interesting properties for a wide variety of applications [3,4,5]. Chitosan hydrogels can also be prepared by the Diels–Alder reaction [6,7,8,9]. Many chitosan derivatives have been developed to improve the encapsulation efficiency of hydrophilic drugs, including chitosan-*g*-poly(ethylene) glycol [10,11]. A wide range of poly(ethylene)glycol (PEGs) with functionalized end groups (e.g., azides, thiols, maleimides, carboxylic acids, hydroxyls, and epoxides) are currently available, increasing the attractiveness of their use in biomedical and biomaterial applications [12]. PEG-based hydrogels have shown interesting properties for their implementation in biological applications such as low toxicity and rapid elimination after oral administration.

An alternative and relatively novel approach to classical derivatization methods is the use of click chemistry reactions. The term click chemistry was first coined by K. B. Sharpless in 2001 to describe the rapid formation of crosslinked networks from modular units [13]. Click chemistry reactions are characterized by high yields and proceed without the formation of by-products. These reactions are stereospecific and thermodynamically favorable. Currently, the use of click chemistry in polymer research is an interesting strategy to design polymer-based hydrogels, drug and gene delivery systems, scaffolds for tissue engineering, among other materials [14,15]. This is due to the advantages offered by these reactions to control the properties of hydrogels, their degree of crosslinking, and in some cases, thermal reversibility.

Among click chemistry reactions, the Diels–Alder (DA) coupling reaction involves a highly selective [4 + 2] cycloaddition between a conjugated diene and a dienophile to form a stable cycloadduct. The Diels–Alder reaction must be considered as a chemical equilibrium, where the direct reaction proceeds at low temperatures (normally below 90 °C) and the reverse reaction that takes place at higher temperatures as a consequence of its higher activation energy [16]. The structural characteristics of furan and its derivatives—together with its natural and renewable origin—make it the ideal dienic reagent for the Diels–Alder reaction [17]. Today, the most studied diene/dienophile system is furan/maleimide, which can react through the Diels–Alder cycloaddition and is, in many cases, reversible at temperatures above 100 °C [18,19].

**Scheme 1:**
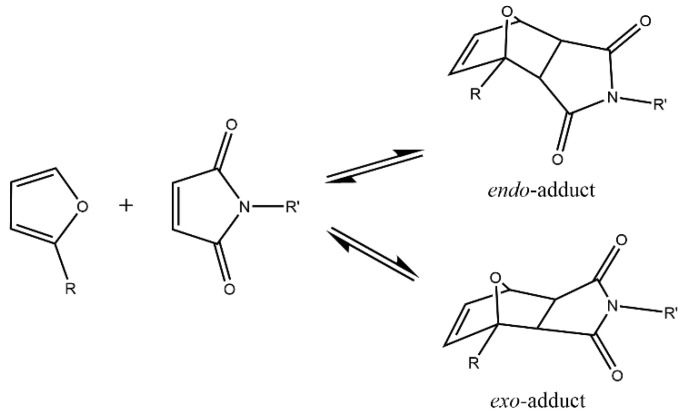
Diels–Alder cycloaddition reaction between furan and maleimide derivatives.

It is well known that the cycloadduct formed during the Diels–Alder reaction has two stereoisomers, *endo* and *exo*. Furans participate in Diels–Alder reactions with dienophiles, producing *exo*-cycloadducts—which are thermodynamically more stable due to less steric hindrance—than their counterpart, *endo*-adducts, which are kinetically favored (Scheme 1).

In this article, the rheological behavior, and aspects of the kinetics of the Diels–Alder crosslinking reaction between *N*-furfuryl chitosan and PEG-maleimide derivatives—which takes place in an aqueous medium—are studied. The influence of temperature, the degree of polymerization, and functionality of PEG-maleimide derivatives on the reaction, as well as the influence of temperature on the mechanical properties of the formed hydrogel, is investigated.

## 2. Materials and Methods

### 2.1. Materials

Chitosan with a degree of acetylation of 18% and weight-average molecular weight of 200 kDa was provided by Primex ehf (Siglufjordur, Iceland). Low polydispersity polyethylene glycol 2- and 4-arm maleimide derivatives were acquired from JenKem Technology, TX, USA. The characteristics of these PEG-maleimide derivatives are summarized in Table 1. All other reagents were purchased from Sigma-Aldrich, Merck KGaA, Darmstadt, Germany. Deionized Type I ultrapure water with a conductivity lower than 0.05 μS/cm was used in all experiments.

### 2.2. Synthesis of N-Furfuryl-Chitosan (FCs)

The synthesis of *N*-furfuryl chitosan was carried out as described by Montiel-Herrera et al. with modifications [6]. Briefly, 2.58 g chitosan (12.17 -NH_2_ mmol) was dissolved in 1000 mL of 2% aqueous acetic acid overnight. Next, 433 μL of furfural (5.23 mmol) was dropped under stirring into the Cs solution at room temperature and allowed to react for 4 h at pH 5. Then, 16 mL of a freshly prepared aqueous solution containing 0.987 g of NaH_3_BCN was slowly added with the aid of a peristaltic pump (Minipuls 3, Gilson, France), and the reaction was further conducted for 3 h. After the completion of the process, the chitosan derivative was precipitated with 1 M NH_4_OH until a pH~7–8 was attained, and successively washed with water until reaching a 0.5 µS/cm conductivity, water:ethanol mixtures 40:60, 20:80 (*v:v*) and ethanol. Finally, the purified sample was dried at room temperature under vacuum (yield: 83%).

### 2.3. Fourier Transformed Infrared Spectroscopy (FTIR)

The spectra were recorded with a Nicolet Protege team (System 460 E.S.P) FT-IR spectrometer (Madison WI, USA) by the accumulation of 64 scans with a resolution of 4 cm^−1^. The attenuated total reflectance (ATR) module was used.

### 2.4. Nuclear Magnetic Resonance (^1^H-NMR)

High-resolution ^1^H-NMR spectroscopy was carried out on a Bruker Avance 400 spectrometer (Billerica, MA, USA) operating at 400 MHz, and 90° pulse was 14.0 ms. The spectra were registered at 25 °C. Samples of FCs were solubilized in D_2_O/DCl (polymer concentration ≈ 6 mg/mL).

### 2.5. Rheological Studies

Dynamic viscoelastic measurements were carried out using an AR-G2 rheometer (TA Instruments, New Castle, DE, USA) equipped with a stainless-steel parallel plate geometry (diameter: 40 mm; gap: 1000 µm) and a Peltier system for temperature control.

A fresh FCs solution prepared the day before in acetic acid (2%) was mixed with the appropriate amount of the maleimide compound and stirred for a few minutes. The mole ratio of maleimide to furan groups, R, was chosen to be 0.25 or 0.5. Before loading the sample into the rheometer, it was centrifuged for 6 min at 15,000× *g*, to remove bubbles. A thin layer of low viscosity silicone oil was added around the periphery of the geometry plate to prevent the evaporation of the sample.

All experiments were performed isothermally, according to the following program: initially, a mechanical spectrum was recorded at 25 °C within a frequency range of ω = 0.1–100 rad·s^−1^, followed by rapid heating of the sample up to the gelation temperature, typically in 30–45 s. The Diels–Alder reaction was then monitored isothermally by the variation of *G*′ and *G*″ at three frequencies between 1 and 10 rad·s^−1^. In all cases, a 2.5% strain was used, thus ensuring that measurements were performed within the linear viscoelastic region and avoiding damages to the gel that is forming. Unless otherwise stated, at the end of the crosslinking process a mechanical spectrum was accomplished at the gelation temperature, followed by another one at 25 °C under the same conditions as described above.

## 3. Results and Discussion

### 3.1. Diels–Alder Click Reaction for Chemical Crosslinking of Chitosan

Diels–Alder cycloaddition is one of the click chemistry reactions, allowing a high process control and valuable versatility. In macromolecular chemistry, it can be used for polymerization, polymer modification, gelation, and thanks to its dynamic character, advanced applications have also been proposed. There are studies in which this reaction is applied for the formation of chemical gels of polysaccharides in particular chitosan, rendering networks with interesting properties.

A synthetic pathway for grafting furan groups into the chitosan backbone under mild conditions with adequate regioselectivity has been proposed (Scheme S1) [6]. The derivative obtained was characterized by FTIR and ^1^H-NMR spectroscopies (Figures S1 and S2, respectively). In the FTIR spectra there are three bands at 746, 821, and 901 cm^−1^ due to the presence of the furan ring on the *N*-furfuryl chitosan. Meanwhile, the NMR spectrum of the derivative shows the characteristic signals of the furan ring between 6.25 and 7.5 ppm. From the latter, a degree of *N*-furfuryl substitution of 21% was estimated.

**Scheme 2:**
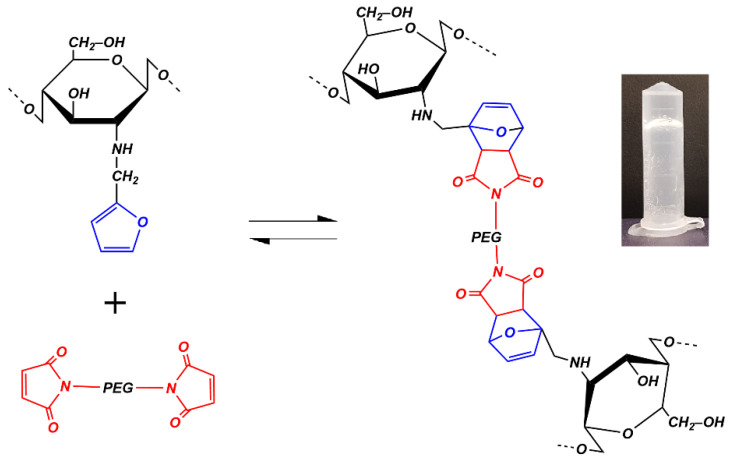
Diels–Alder reaction between *N*-furfuryl chitosan and PEG-maleimide compounds.

In the present study, polyethylene glycol maleimide-terminated derivatives with different degrees of polymerization and functionalities were chosen (Table 1). The Diels–Alder reaction between *N*-furfuryl chitosan and these PEG-maleimide derivatives gives clear and transparent hydrogels, with excellent mechanical properties, as can be appreciated in Scheme 2.

The FTIR spectra of the *N*-furfuryl chitosan, PEG-bismaleimide, and the Diels–Alder adduct hydrogel are presented in Figure S3. The bismaleimide compound exhibits the characteristic bands of poly(ethylene glycol)s and maleimide groups, particularly the bands at 1707 cm^−1^ (C=O stretching), 1466 cm^−1^ (C=C stretching), 1341 cm^−1^ (C–N stretching), 1150 cm^−1^ (C–N–C bending), 840 cm^−1^ (C–H wagging vibration), and 695 cm^−1^ (=C–H out-of-plane bending) [20,21,22]. The chitosan derivative, among others, displays the signals corresponding to the furan ring: 1374 cm^−1^ (C=C furan ring stretching vibration), 895 cm^−1^ (out-of-plane deformation vibration), and 660 cm^−1^ (furan ring deformation). After the crosslinking Diels–Alder reaction, it is evident that maleimide and furan characteristic bands are all diminished in the adduct spectrum, confirming the consumption of the maleimide and furan groups. Moreover, there are new peaks at 1454 cm^−1^ (C=C stretching), 1258 cm^−1^ (C–O symmetric stretching), 947 cm^−1^ (C–H in-plane deformation), and 799 cm^−1^ (C–H out-of-plane deformation) [8,23,24]. These results confirm the crosslinking Diels–Alder reaction between *N*-furfuryl chitosan and PEG-bismaleimide, and the formation of the cycloadduct.

The hydrogel system formed by *N*-furfuryl chitosan (1%-*w*/*w*), PEG(mal)_2_-7500, and a mole ratio of maleimide to furan groups of 0.25 was first selected. The dynamic mechanical spectra at 25 °C of the precursor solution, and that of the corresponding hydrogel prepared at 50 °C, are displayed in Figure S4. The viscoelastic behavior of the reaction mixture matches that of a concentrated polymer solution, with both *G*′ and *G*″ showing frequency dependence (Figure S4a). Moreover, the frequency sweep of the resulting hydrogel is the typical mechanical spectrum of a crosslinked polymer system (Figure S4b): the storage modulus has no dependence on the frequency and is almost two orders of magnitude higher than *G*″. This corroborates that the Diels–Alder reaction gives rise to a hydrogel with good mechanical properties, as it will be confirmed later.

### 3.2. Rheological Analysis of the Influence of Temperature on Diels–Alder Cycloaddition for Chemical Crosslinking

Among the different experimental parameters having a major effect on the Diels–Alder click reaction, temperature is of the utmost importance. Indeed, the Diels–Alder cycloaddition is, in fact, an equilibrium chemical process. The relationship between Diels–Alder and retro-Diels–Alder reactions determines many of their applications, as well as the mechanical properties of the resulting hydrogels.

The kinetics of the gelation process and the viscoelastic properties of the hydrogels prepared via the DA reaction between *N*-furfuryl chitosan and PEG-maleimide derivatives were then investigated by low deformation dynamic rheology, under isothermal conditions at temperatures between 50 and 90 °C.

**Figure 1:**
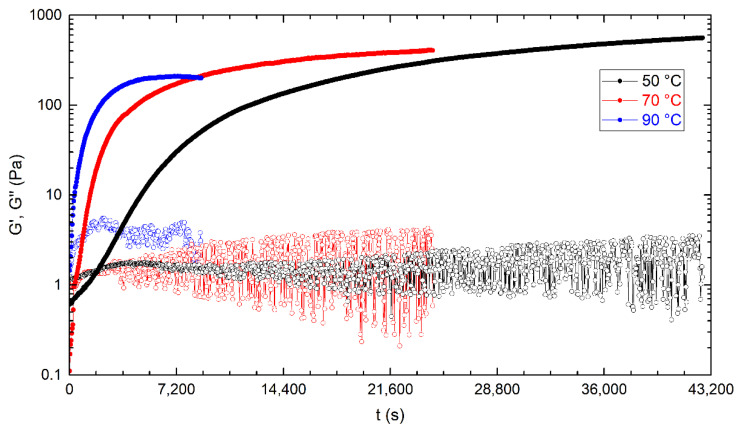
Evolution of storage modulus (*G*′), and loss modulus (*G*″) with time for the system *N*-furfuryl-chitosan (21% substitution degree) and PEG(mal)_2_-7500. Polymer concentration: 1 wt.%, and R = 0.25. *G*′, closed symbols, and *G*″, open symbols. Only traces for ω = 1 rad·s^−1^ are shown.

In Figure 1, the evolution of the storage and loss moduli during the gelation at 50, 70, and 90 °C is presented. It is evident that, as the temperature increases, the time necessary to reach equilibrium decreases from 12 to around 2 h, while the equilibrium values of the storage modulus also diminish.

Polymeric materials are characterized by broad relaxation spectra, reflecting the variety of polymeric species present. Before the gel point, a crosslinking system exhibits increasingly slower movements as the polymeric crosslinked clusters increase in size, which is accompanied by an increase in their viscosity values. Near the critical point, the relaxation time grows sharply, the relaxation modes are no longer independent, and a characteristic relaxation time cannot be identified anymore. The gel point is reached when the largest molecular group diverges to a theoretical infinite size, limited by the volume of the reactor.

At the critical gelation point, the shear flow properties under steady flow conditions are a sensitive indicator of the proximity of the gel point. The viscosity of the sol fraction increases due to the size of the divergent cluster. The equilibrium modulus gradually increases as an increasing fraction of the molecules binds and thus the sample spanning the entire permanent lattice is strengthened.

A common condition used to determine the isothermal gel time by rheology was suggested by Tung and Dynes as the *G*′–*G*″ crossover point, *tan*(*δ*) = *G*″/*G*′ = 1 [25]. However, in multi-frequency tests, this crossover point is frequency dependent, and it is obvious that the gel point cannot depend on the frequency, as in our system (e.g., Figure S5).

Instead, Winter and Chambon have proposed another criterion, considering that at the critical gel point the system is neither in a viscous liquid state nor an elastic network [26]. Before the gel point, the medium can be considered as a liquid for which *G*′~ *ω*^2^ and *G*″~ *ω*. As the crosslinking reaction progresses, there is an instant—the critical gel point—at which *G*′ and *G*″ exhibit a power-law dependence with frequency, *ω*, of the type *G*′(*ω*)~ *G*″(*ω*)~ *ω^n^*.

(1) $$tan\delta \left(\omega \right)=\frac{G_{c}^{\prime \prime }}{G_{c}^{\prime }}=tan\frac{n\pi }{2}$$

In this case, “*n*” is the critical relaxation exponent [27]. After the gel point, the reaction medium is considered equivalent to an elastic solid.

That means that at the critical gel point the *tan*(*δ*) is independent of frequency, so in a multi-frequency experiment, the *tan*(*δ*) curves pass through a single point. Thus, during a dynamic viscoelastic experiment, simultaneous measurement of *tan*(*δ*) at several frequencies provides a convenient method of interpolation for determining the gel point, which is known as the Winter–Chambon criterion. This is under the condition that the deformation remains within the limits of linear viscoelasticity, and the stress is so low, as to avoid any chance of breaking the delicate network in its incipient stages [28].

**Figure 2:**
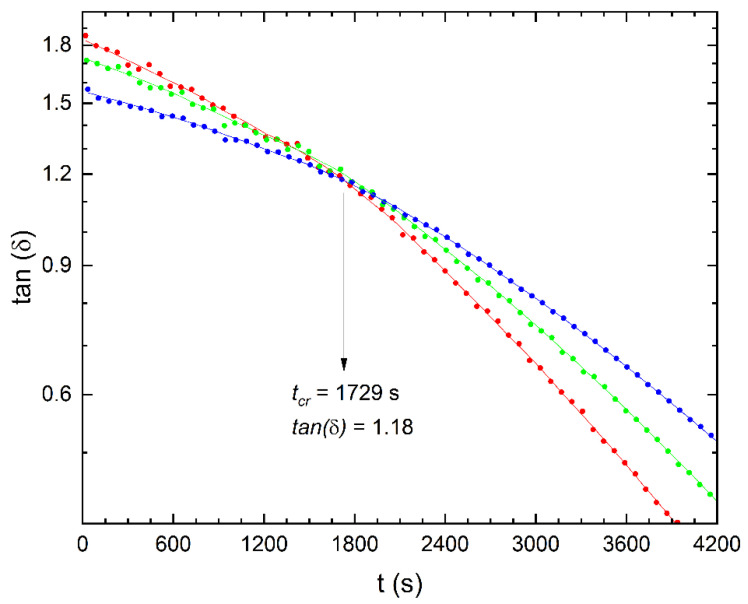
The loss tangent, *tan*(*δ*), as a function of the reaction time during the Diels–Alder click reaction between *N*-furfuryl chitosan (21% degree of substitution) and PEG(mal)_2_-7500 at 50 °C. The *tan*(*δ*) curves at different frequencies crossover at the gel point (ω = 2.15, 4.64 and 10 rad·s^−1^; red, green, and blue, respectively). Polymer concentration: 1 wt.%, and R = 0.25.

Applying this approach, it was possible to assess the critical gel times for this hydrogel at temperatures between 50 and 90 °C. The estimation of the gel time at 50 °C is exemplified in Figure 2. According to the Winter and Chambon criterion, *n*, the critical relaxation exponent, equals 0.552. Values of *n* larger than 0.5 have been evaluated for stoichiometrically imbalanced gels, characterized with a crosslinker deficiency as in this case [29]. The critical relaxation exponent is known to be independent of temperature [30].

The values of the critical gel time for all temperatures, as well as those of the gel strength, S, are summarized in Table 2. The latter is a parameter depending on the material structure at the transition state and affects the linear constitutive viscoelastic equation. It could be estimated from *G*′ at the intersection with *G*″, and it is accepted that it varies with temperature [26,30,31]. As it can be appreciated, the reaction proceeds faster as the temperature increases up to 90 °C, while the strength of the gel decreases under the same conditions.

According to the theory of Winter and Chambon, the gel time should be interpreted as the time at which the critical sol-to-gel conversion occurs during chemical crosslinking under isothermal conditions. Gel times are an intrinsic property of the material at the critical gel point, in the sense that they do not depend on the method, but on the specific characteristics of the polymeric system, under the experimental conditions in which the process takes place. It is reasonable, therefore, to assume that—under the condition that there is only one reaction mechanism involved—the gel time can be considered a quantitative measure of the rate of the overall reaction [32].

From the previous discussion, it could be understood that the gel time adequately reflects the kinetics of a chemical crosslinking process, such as the one that concerns us in this study. It is then possible to develop the following mathematical considerations on the kinetics of chemical gelation [32,33,34]. Given the concentration of each type of functional groups, *C*, the rate constant, *k*, and the kinetic order, *n*, the general equation for the reaction rate is expressed as:

(2) $$-\frac{dC}{dt}=kC^{n}$$

Since we are interested in the kinetic expression at the gel point, Equation (2) should be integrated. The concentration of functional groups at the beginning of the reaction, *t*_0_, and at the gel point, *t_gel_*, will be labeled as *C*_0_ and *C_gel_*, respectively.

(3) $$kt_{gel}=\left[\frac{1}{\left(n-1\right)}\right]\left(C_{gel}^{1-n}-C_{0}^{1-n}\right)$$

The term on the right is a constant, and will be set equal to *B*, which makes the rate constant equivalent to $k=B/t_{gel}$. Being an expression of the kinetics of the reaction, its dependence on temperature obeys the Arrhenius equation with a dependence of this type:

(4) $$k=\frac{B}{t_{gel}}=Ae^{-\frac{E_{a}}{RT}}$$

where *A* is the pre-exponential factor, and *R* and *T* have their usual significance. Applying logarithms and rearranging, the following expression is attained:

(5) $$\ln t_{gel}=\ln\frac{B}{A}+\frac{E_{a}}{RT}$$

which allows for a straightforward evaluation of the activation energy from the values of the gel time on isothermal experiments at different reaction temperatures.

This kinetic analysis should be valid under initial conditions in which the direct Diels–Alder reaction is the predominant one. The experimental conditions that must prevail before the critical gel point—particularly in aqueous media and low temperatures, compared to those of other systems—are not logical to be affected by the retro-Diels–Alder reaction since it would be denying the very fact of gelation. Beyond the gel point, when the mobility of the reacting species is severely restricted, it can be speculated that the probabilities of the reverse reaction will increase, affecting the mechanical properties of the final hydrogel.

**Figure 3:**
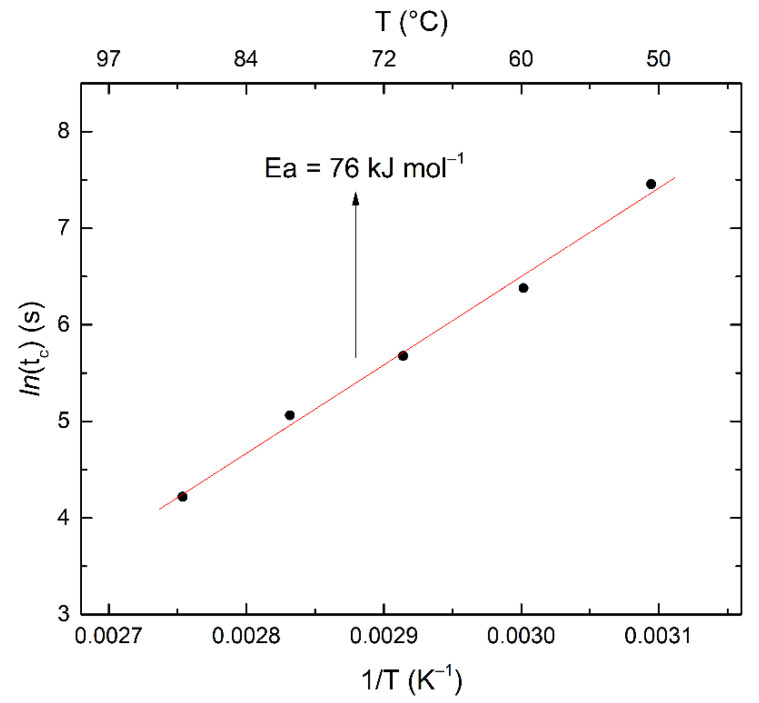
Arrhenius plot of the logarithm of the gelation time vs. reaction temperature according to Equation (5). Hydrogels formed between *N*-furfuryl chitosan (1%-*w*/*w*) and PEG(mal)_2_-7500 with R = 0.25. All experimental parameters as specified in Figure 2.

Figure 3 depicts the dependence of gel time on temperature. It is evident that good correlation is attained within the temperature range under study. This result could be indicating that, at least under these experimental conditions, the retro-Diels–Alder reaction does not appear to be affecting the kinetics of the chemical crosslinking process as evidenced by rheological means. From this plot, the activation energy was calculated to be 76 kJ·mol^−1^. This value is slightly higher than those found for similar systems by other authors. Normally, values between 30 and 55 kJ·mol^−1^ have been reported [22,35,36,37,38,39,40]. This wide range of values in the activation energy could be explained by the specific differences between the chosen diene/dienophile, as well as by other important experimental conditions, such as the solvent or the bulk reaction. To our knowledge, the highest value that has been obtained is 67 kJ·mol^−1^, for the bulk maleimide–furan DA reaction assuming second-order kinetics [41].

The present investigation was carried out in a dilute aqueous solution of acetic acid. It is known that the Diels–Alder reaction is considerably faster in water than in organic solvents [42,43,44]. Although there is still no single explanation, it is considered that this effect is a consequence of the polarity of the medium, and the “enforced hydrophobic interactions”, generating a packing of the diene and dienophilic groups. Hydrogen bond interactions also contribute to this acceleration. What is more significant in this case, however, is the role of solvent and temperature in the resulting ratio of the two stereoisomers, although here are some conflicting data as well [43].

In 1952 Kwart reported that, in the reaction of maleimide with furan, regardless of the solvent used (water or ether), at 25 °C the *endo*-isomer is favored, but at 90 °C the *exo*-adduct is formed [45]. The use of high temperatures allows dissociation and recombination of the adduct, leading to the formation of the thermodynamically stable (*exo*) adduct, at the expense of the kinetically favored isomer (Scheme 1) [46]. At the same time, reports are indicating that water and other strongly polar solvents cause a loss in the aromaticity of furan, causing the reaction to be thermodynamically controlled (*exo*-adduct formation) [47]. In the specific case of the reaction of furfural with maleimide derivatives in water (60 °C), *exo*-adducts are preferably formed in all cases [48].

At the same time, recent kinetic studies on the stereochemistry of the furan/maleimide DA reaction reveal that the *endo*-isomer has a lower activation energy value than the *exo*-isomer [49,50,51].

On the one hand, a higher value of activation energy would suggest that, under our experimental conditions, the *exo*-adduct is being formed preferentially. Nevertheless, the effect of diffusion constraints on the mobility of the polymer chains during the progressive crosslinking, cannot be ruled out either [52]. The former suggests the formation of *exo*-isomers, while the latter would explain why a reaction with higher activation energy—requiring more thermal activation—is favored if it is kinetically disfavored [53,54].

### 3.3. Viscoelastic Characterization of the Hydrogels Obtained at Different Temperatures

**Figure 4:**
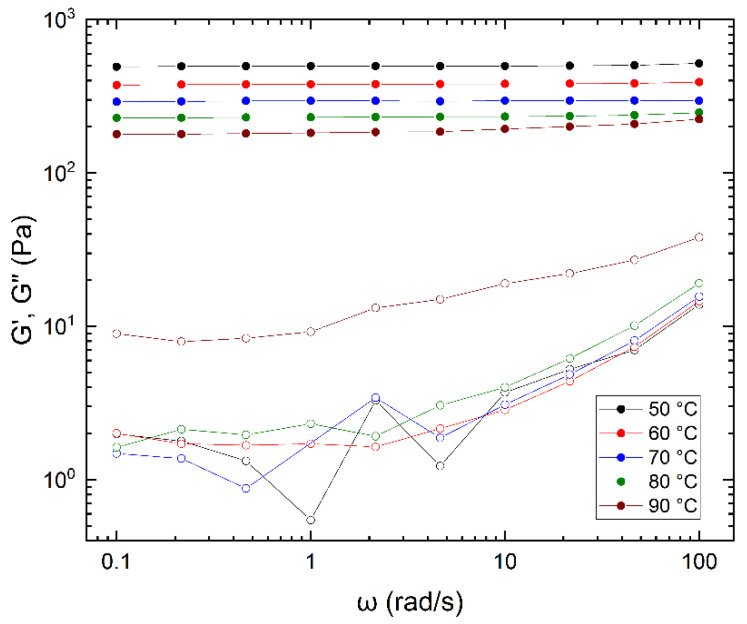
Frequency sweeps of the system of *N*-furfuryl chitosan 1 wt.% (21% degree of substitution) in 2% acetic acid and PEG(mal)_2_-7500 with R = 0.25 prepared at 50, 60, 70, 80, and 90 °C, as indicated in the figure. All the measurements were conducted at 25 °C and γ = 2.5%. *G*′, closed symbols, and *G*″, open symbols.

A general outlook of the viscoelastic properties of the hydrogels prepared at temperatures between 50 and 90 °C appears in Figure 4 (regardless of the gelation temperature, all mechanical spectra were recorded at 25 °C for comparison purposes). At first glance, it should be noted that, by varying the temperature of the Diels–Alder reaction, hydrogels with good mechanical properties are obtained. The storage modulus is independent of the frequency and shows values between 200 and 500 Pa. The loss modulus, on the other hand, exhibits values two orders of magnitude lower than G’, except for the material formed at 90 °C, which is the weakest of the series.

As was already outlined in Figure 1 and Table 2, the elastic modulus follows a tendency to diminish as the gelation temperature increases. This effect does not seem to be an experimental artifact, nor a consequence of the retro-Diels–Alder reaction. The latter should be ruled out as the kinetics of the process does not shed light on the existence of this reaction under the employed experimental conditions.

This behavior seems to be a consequence of the reaction kinetics itself because as the crosslinking between the polymer chains takes place faster, the mobility of the polymer segments freezes earlier, and the possibilities of reaction between complementary functional groups become scarcer. This phenomenon is also evident from the very rapid evolution of the storage modulus at 90 °C, compared to that observed at 50 °C (Figure 1). Therefore, the lower the crosslinking density, the weaker the elastic response of the material should be.

To validate this hypothesis, the crosslinking density and the network mesh-size of these hydrogels were calculated. For this purpose, if we assume that the frequency sweeps were carried out under linear viscoelastic conditions—and it is reasonable given the experimental conditions used—then it is possible to estimate the crosslinking density from the basic concepts of Flory’s theory of elasticity. Then, the crosslinking density, $\rho_{x}$, was evaluated by the following equations [55]:

(6) $$\rho_{x}=\frac{G^{\prime }}{RT}$$

while the network average mesh-size, $\xi_{a}$, becomes:

(7) $$\xi_{a}=\sqrt[{3}]{\frac{6}{\pi \rho_{x}N_{a}}}$$

where *R*, *T*, and $N_{a}$ are the universal gas constant, the absolute temperature, and the Avogadro Number, respectively.

From the average values of the equilibrium storage moduli shown in Figure 4, the crosslinking density and network mesh-sizes values were estimated and presented in Table 3.

Indeed, the values of the crosslinking density from Table 3 confirm that this parameter decreases as the reaction temperature increases. At high temperatures, crosslinking is rapid, which “freezes” the segmental motions of the chains, thus preventing a closer approach between functional groups. Consequently, a lower reaction completion and slightly weaker mechanical properties were observed. In other words, the crosslinking density and the elastic properties of the hydrogel seem to be controlled by the diffusion of the polymer segments, rather than by the kinetics of the Diels–Alder reaction. The possible influence of the reverse reaction at higher temperatures once the gel is maintained at these temperatures after the gel point cannot be ruled out either.

### 3.4. Influence of Other Eexperimental Parameters: Concentration of Chitosan, Maleimide to Furan Molar Ratio, and Characteristics of the Maleimide Crosslinker

**Figure 5:**
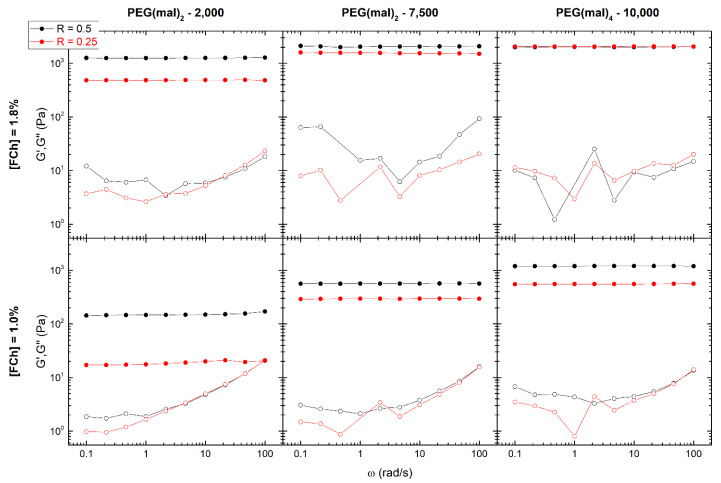
Mechanical spectra of *N*-furfuryl chitosan hydrogels based on the Diels–Alder click reaction varying the degree of polymerization and functionality of the PEG-maleimide compound, as indicated in the figure. Frequency sweeps were performed at 25 °C and γ = 2.5%, after gelation at 70 °C. *N*-furfuryl chitosan (21% degree of substitution) dissolved in 2% acetic acid. Polymer concentration: 1.0 and 1.8 wt.%, and R = 0.25 (red) and 0.5 (black), as indicated in the figure. *G*′, closed symbols, and *G*″, open symbols.

After analyzing the influence of reaction temperature, some other important experimental parameters were considered as well. Figure 5 summarizes the mechanical spectra registered at 25 °C, of hydrogels prepared at 70 °C, varying the following parameters: (i) polymer concentration: 1.0 and 1.8 wt.%; (ii) maleimide to furan molar ratio: 0.25 and 0.5; and (iii) characteristics of the maleimide crosslinker: degree of polymerization and functionality (as specified in Table 1). From a first general analysis of their mechanical spectra, it is evident that in all cases (except for the hydrogels prepared with a concentration of FCh 1% and PEG(mal)_2_-2000), the storage modulus presents values greater than 200 Pa, reaching a limit of 2000 Pa.

Following the same calculation procedure as for the hydrogels prepared at different temperatures, the crosslinking density was also calculated using Equation (6), and the results obtained are summarized in Table 4.

As expected, by increasing the concentration of chitosan there is a reinforcement of the mechanical properties, regardless of the value of R employed. The same happens when comparing the maleimide:furan molar ratio, at equal chitosan concentrations. In both cases, an increase in the concentration of any of the two functional groups is accompanied by a higher crosslinking density (Table 4). A similar effect has been reported during the chemical crosslinking of furan/maleimide system with hyaluronic acid, and semidiluted solutions of poly(vinyl alcohol) [8,56].

The role played by the degree of polymerization of polyethylene glycol on the hydrogel characteristics is interesting. For bifunctional maleimide derivatives, it is evident that an increase in chain length (DP: 45 vs. 170) has a noticeable effect on the gel. It can be speculated that due to their remarkable hydrophilicity and chain flexibility, PEGs have a significant impact on reaction completion by allowing better diffusion of maleimide groups in the reaction medium. This effect is more important at low concentrations of chitosan, whose chains are notably more rigid.

The effect of the functionality of the maleimide crosslinker deserves separate mention. As expected, the introduction of a tetrafunctional PEG-maleimide derivative leads to an increase in the storage modulus, accompanied by a higher crosslinking density.

### 3.5. Influence of the Temperature on the Mechanical Properties of a Diels–Alder Hydrogel

**Figure 6:**
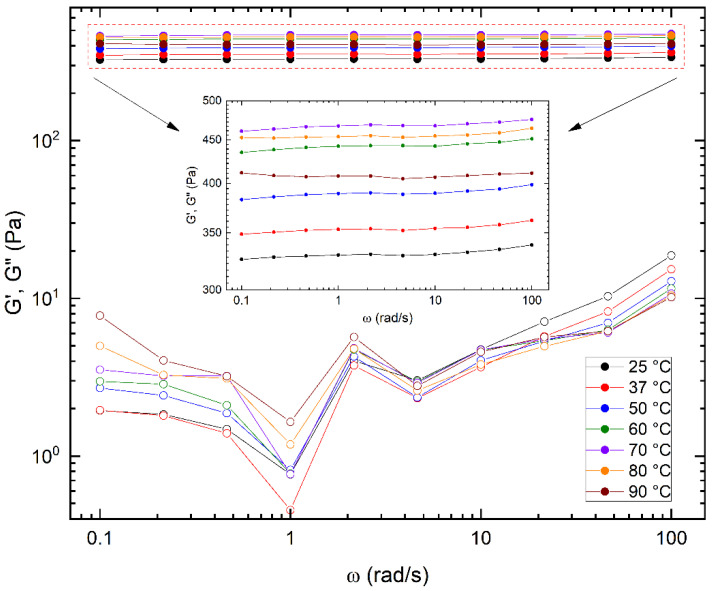
Mechanical spectra of *N*-furfuryl chitosan hydrogels based on the Diels–Alder click reaction after gelation at 70 °C. Frequency sweeps were performed at 25, 37, 50, 60, 70, 80, and 90 °C as indicated in the figure. The dependence of the storage modulus with angular frequency is magnified inside the figure. *N*-furfuryl chitosan 1-wt.% (21% degree of substitution) in 2% acetic acid, PEG(mal)_2_-7500, R = 0.25, and γ = 2.5%. *G*′, closed symbols, and *G*″, open symbols.

The effect of the temperature on the mechanical properties of a Diels–Alder crosslinked hydrogel was also investigated. For this purpose, a hydrogel was prepared with *N*-furfuryl chitosan 1.0 wt.% and PEG(mal)_2_-7500, R = 0.5 at 70 °C. Once the hydrogel was formed, after 5 h of reaction, the temperature was lowered to 25 °C, and a frequency sweep was recorded after 15 min of equilibration. Then, the temperature was successively raised to 37, 50, 60, 70, 80, and 90 °C, and a mechanical spectrum registered after equilibration at the corresponding temperature for 15 min. The results of the thermorheological behavior of the hydrogel are displayed in Figure 6 (the dependence of the storage modulus with angular frequency is magnified inside the figure).

**Figure 7:**
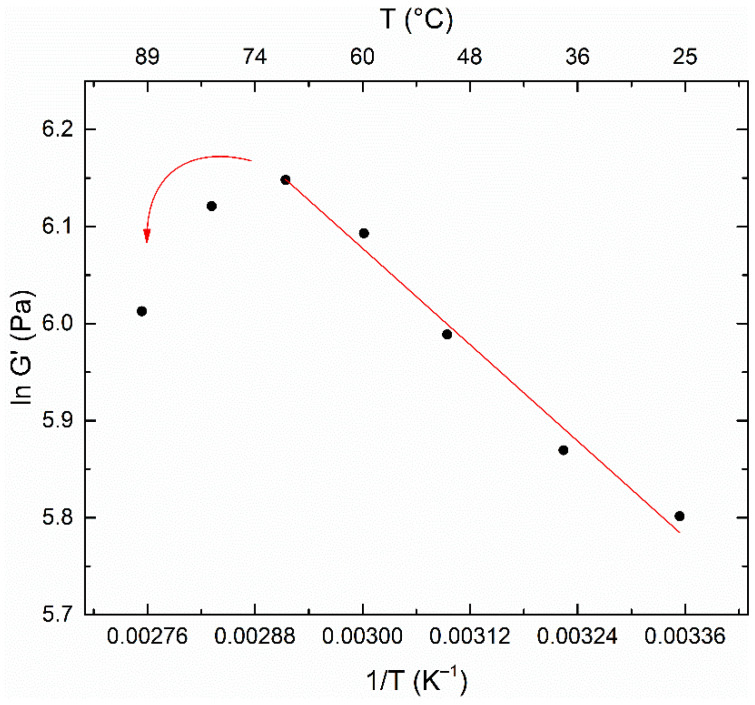
Arrhenius plot of the logarithm of the storage modulus vs. temperature. All experimental parameters as specified in Figure 6.

In the inset of Figure 6, a slight variation in the *G*′ values is observed as the temperature of the hydrogel increases. To obtain a clear view about the dependence of this parameter on temperature, the average values of the equilibrium storage modulus were plotted on an Arrhenius-type plot, which are shown in Figure 7.

In Figure 7 it is interesting to note a strengthening of the elastic characteristics of the hydrogel as it is heated from 25 to 70 °C, resulting in a strong linear Arrhenius-type dependence. The origin of this behavior is not clear, but it could be due to an increase in the completeness of the Diels–Alder reaction. It should be noted that an Arrhenius-like behavior is the overall result of interactions between macromolecules and conformational changes, which inevitably leads to the dissociation and reformation of the crosslinks. The latter reflects the behavior of a system with dynamic crosslinking bonds, as in the case of the Diels–Alder and retro-Diels–Alder reactions [57]. In other words, the hydrogel crosslinked by Diels–Alder cycloadducts is capable of self-completion, increasing the storage modulus (from 330 to 470 Pa) and the crosslinking density (from 1.3 to 1.5 × 10^−7^ mol/cm^3^).

At temperatures above 70 °C, there is a drop in *G*′ values. This deviation from the Arrhenius-like behavior unequivocally indicates that there is a process opposing to the Diels–Alder reaction, most likely the retro-Diels–Alder. Both the Diels–Alder and retro-Diels–Alder reactions proceed at different rates and their equilibrium is strongly influenced by temperature [37]. The hydrogel—which is already crosslinked—can reach an equilibrium between the direct and inverse reactions. That is, the dynamic character of this reaction allows certain rearrangement of the crosslinks within the hydrogel to take place. As a consequence, at temperatures below 80 °C the formation of the cycloadduct is likely to be favored, whereas above 80 °C, the breakdown of the adducts becomes increasingly important.

It should be mentioned that the actual difference in the values of the storage modulus during the heating of the hydrogel from 25 to 90 °C is very small (330–467 Pa), and therefore the variation in the degree of crosslinking is not large, as it would be expected for such a dynamic rearrangement of the polymer network.

These dynamic features of the Diels–Alder equilibrium underlie the self-healing and remendability behavior of these hydrogels. They represent a challenging and rapidly developing scientific frontier in polymeric materials science.

## 4. Conclusions

Clear and transparent hydrogels with excellent mechanical properties were prepared between *N*-furfuryl chitosan and PEG-maleimide derivatives in dilute aqueous acidic conditions. Applying the Winter and Chambon criterion, the gel times were estimated at different temperatures, and the activation energy was calculated to be 76 kJ·mol^−1^. This value suggests that, under our experimental conditions, the *exo*-adduct is being formed preferentially.

The elastic modulus of the hydrogels prepared at temperatures between 50 and 90 °C follows a tendency to diminish as the gelation temperature increases. At high temperatures, crosslinking is rapid, which “freezes” the segmental motions of the chains; thus, preventing a closer approach between functional groups and the possibilities of reaction between complementary functional groups become scarce. The possible influence of the reverse reaction at higher temperatures once the gel is maintained at these temperatures after the gel point cannot be ruled out either.

The role played by the degree of polymerization of the crosslinking PEG-maleimide derivatives on the hydrogel mechanical properties is interesting. For bifunctional maleimide derivatives, it is evident that an increase in chain length of PEG has a noticeable effect on the strength of the gel. It can be hypothesized that, due to their remarkable hydrophilicity and chain flexibility, PEGs chains display an important effect on reaction completion by allowing a better diffusion of the maleimide groups in the reaction medium. This effect is more important at low concentrations of chitosan, whose chains are notably more rigid.

Once obtained, the hydrogel showed an improvement in its mechanical properties as the temperature increases up to 70 °C. Above this temperature, a decrease in the storage modulus is observed, indicating the existence of a process opposite to the Diels–Alder reaction, most likely the retro-Diels–Alder.

## Figures and Tables

**Table 1:** Identification and characteristics of the poly(ethylene)glycol -maleimide derivatives.

Identification	*f* ^1^	Substitution ^2^ (%)	Molecular Weight (Polydispersity) ^3^	Purity ^4^ (%)
PEG(mal)_2_-2000	2	97.9	2084 (1.04)	99.2
PEG(mal)_2_-7500	2	98.1	7408 (1.03)	100
PEG(mal)_4_-10,000	4	94.7	10,160 (1.04)	98.0

^1^ *f*: functionality. ^2^ Determined by ^1^H NMR, reported by the supplier. ^3^ Molecular weights (polydispersity) by MALDI GPC), reported by the supplier. ^4^ Determined by HPLC, reported by the supplier.

**Table 2:** Critical gel times and the strength of the hydrogel at gelation for the system between *N*-furfuryl chitosan (1%-*w*/*w*) and PEG(mal)_2_-7500 with R = 0.25.

T_gel_ (°C)	t_gel_ (s)	S (Pa s^1/2^)
50	1729	1.15
60	590	0.94
70	292	0.87
80	158	0.75
90	68	-

**Table 3:** Values of crosslinking density, *ρ_x_*, and network mesh-size values, *ξ_a_*, of the hydrogels, calculated from Equations (6) and (7), respectively. All experimental parameters as specified in Figure 4.

T_gel._ (°C)	G’ (Pa)	*ρ_x_* (mol∙cm^−3^)	*ξ_a_* (nm)
50	499.8	2.02 × 10^−7^	25
60	380.5	1.53 × 10^−7^	27
70	294.7	1.19 × 10^−7^	30
80	233.7	0.94 × 10^−7^	32
90	191.9	0.77 × 10^−7^	34

**Table 4:** Values of crosslinking density, *ρ_x_*, calculated from Equation (6) for hydrogels based on the Diels–Alder click reaction at different degrees of polymerization and functionalities of the PEG-maleimide compound. To keep the visual association with the curves in Figure 5, the black and red colors are maintained.

		PEG(mal)_2_-2000	PEG(mal)_2_-7500	PEG(mal)_4_-10,000
[FCs] (wt.%)	R	Crosslinking Density (mol/cm^3^)		
1.8	0.5	5.09 × 10^−7^	8.33 × 10^−7^	8.17 × 10^−7^
	0.25	1.97 × 10^−7^	6.28 × 10^−7^	8.34 × 10^−7^
1.0	0.5	0.61 × 10^−7^	2.28 × 10^−7^	4.84 × 10^−7^
	0.25	0.07 × 10^−7^	1.19 × 10^−7^	2.24 × 10^−7^

* All experimental parameters as specified in Figure 5.

## Data Availability

Not applicable.

## Supplementary Materials

The following are available online at https://www.mdpi.com/article/10.3390/polym14061202/s1, Scheme S1: Synthesis of FCs; Figure S1: FT-IR spectra of chitosan and FCs; Figure S2: ^1^H-NMR spectrum of FCs; Figure S3: FTIR spectra of the *N*-furfuryl chitosan, PEG-bismaleimide, and the Diels–Alder cycloadduct; Figure S4: Dynamic mechanical spectra at 25 °C of the precursor solution, and that of the corresponding hydrogel prepared at 50 °C; Figure S5: Storage and loss moduli as a function of the reaction time of the gelation process.

## References

[1] Kumirska J., Weinhold M.X., Thöming J., Stepnowski P. (2011). Biomedical Activity of Chitin/Chitosan Based Materials—Influence of Physicochemical Properties Apart from Molecular Weight and Degree of N-Acetylation. Polymers.

[2] Kim S. (2018). Competitive Biological Activities of Chitosan and Its Derivatives: Antimicrobial, Antioxidant, Anticancer, and Anti-Inflammatory Activities. Int. J. Polym. Sci..

[3] Argüelles-Monal W., Goycoolea F.M., Peniche C., Higuera-Ciapara I. (1998). Rheological Study of the Chitosan/Glutaraldehyde Chemical Gel System. Polym. Gels Netw..

[4] Moura M.J., Figueiredo M.M., Gil M.H. (2007). Rheological Study of Genipin Cross-Linked Chitosan Hydrogels. Biomacromolecules.

[5] Espinosa-García B.M., Argüelles-Monal W.M., Hernández J., Félix-Valenzuela L., Acosta N., Goycoolea F.M. (2007). Molecularly Imprinted Chitosan–Genipin Hydrogels with Recognition Capacity toward O-Xylene. Biomacromolecules.

[6] Montiel-Herrera M., Gandini A., Goycoolea F.M., Jacobsen N.E., Lizardi-Mendoza J., Recillas-Mota M., Argüelles-Monal W.M. (2015). N-(Furfural) Chitosan Hydrogels Based on Diels–Alder Cycloadditions and Application as Microspheres for Controlled Drug Release. Carbohydr. Polym..

[7] Montiel-Herrera M., Gandini A., Goycoolea F.M., Jacobsen N.E., Lizardi-Mendoza J., Recillas-Mota M.T., Argüelles-Monal W.M. (2015). Furan–Chitosan Hydrogels Based on Click Chemistry. Iran. Polym. J..

[8] Nimmo C.M., Owen S.C., Shoichet M.S. (2011). Diels−Alder Click Cross-Linked Hyaluronic Acid Hydrogels for Tissue Engineering. Biomacromolecules.

[9] Guaresti O., García–Astrain C., Palomares T., Alonso–Varona A., Eceiza A., Gabilondo N. (2017). Synthesis and Characterization of a Biocompatible Chitosan–Based Hydrogel Cross–Linked via ‘Click’ Chemistry for Controlled Drug Release. Int. J. Biol. Macromol..

[10] Papadimitriou S.A., Achilias D.S., Bikiaris D.N. (2012). Chitosan-g-PEG Nanoparticles Ionically Crosslinked with Poly(Glutamic Acid) and Tripolyphosphate as Protein Delivery Systems. Int. J. Pharm..

[11] Natesan S., Pandian S., Ponnusamy C., Palanichamy R., Muthusamy S., Kandasamy R. (2017). Co-Encapsulated Resveratrol and Quercetin in Chitosan and Peg Modified Chitosan Nanoparticles: For Efficient Intra Ocular Pressure Reduction. Int. J. Biol. Macromol..

[12] Casettari L., Vllasaliu D., Castagnino E., Stolnik S., Howdle S., Illum L. (2012). PEGylated Chitosan Derivatives: Synthesis, Characterizations and Pharmaceutical Applications. Prog. Polym. Sci..

[13] Kolb H.C., Finn M.G., Sharpless K.B. (2001). Click Chemistry: Diverse Chemical Function from a Few Good Reactions. Angew. Chem. Int. Ed..

[14] Lahann J. (2009). Click Chemistry for Biotechnology and Materials Science.

[15] Han J., Wang X., Liu L., Li D., Suyaola S., Wang T., Baigude H. (2017). “Click” Chemistry Mediated Construction of Cationic Curdlan Nanocarriers for Efficient Gene Delivery. Carbohydr. Polym..

[16] Evans D.A., Johnson J.S., Jacobsen E.N., Pfaltz A., Yamamoto H. (1999). Diels-Alder Reactions. Comprehensive Asymmetric Catalysis.

[17] Gandini A., Lacerda T.M. (2015). From Monomers to Polymers from Renewable Resources: Recent Advances. Prog. Polym. Sci..

[18] Gandini A. (2005). The Application of the Diels-Alder Reaction to Polymer Syntheses Based on Furan/Maleimide Reversible Couplings. Polímeros Ciênc. Tecnol..

[19] Gandini A. (2013). The Furan/Maleimide Diels–Alder Reaction: A Versatile Click–Unclick Tool in Macromolecular Synthesis. Prog. Polym. Sci..

[20] Socrates G. (2001). Infrared and Raman Characteristic Group Frequencies: Tables and Charts.

[21] Orozco F., Niyazov Z., Garnier T., Migliore N., Zdvizhkov A.T., Raffa P., Moreno-Villoslada I., Picchioni F., Bose R.K. (2021). Maleimide Self-Reaction in Furan/Maleimide-Based Reversibly Crosslinked Polyketones: Processing Limitation or Potential Advantage?. Molecules.

[22] Liu X., Du P., Liu L., Zheng Z., Wang X., Joncheray T., Zhang Y. (2013). Kinetic Study of Diels–Alder Reaction Involving in Maleimide–Furan Compounds and Linear Polyurethane. Polym. Bull..

[23] Goussé C., Gandini A. (1999). Diels–Alder Polymerization of Difurans with Bismaleimides. Polym. Int..

[24] García-Astrain C., Gandini A., Coelho D., Mondragon I., Retegi A., Eceiza A., Corcuera M.A., Gabilondo N. (2013). Green Chemistry for the Synthesis of Methacrylate-Based Hydrogels Crosslinked through Diels–Alder Reaction. Eur. Polym. J..

[25] Tung C.-Y.M., Dynes P.J. (1982). Relationship between Viscoelastic Properties and Gelation in Thermosetting Systems. J. Appl. Polym. Sci..

[26] Winter H.H., Chambon F. (1986). Analysis of Linear Viscoelasticity of a Crosslinking Polymer at the Gel Point. J. Rheol..

[27] Chambon F., Winter H.H. (1987). Linear Viscoelasticity at the Gel Point of a Crosslinking PDMS with Imbalanced Stoichiometry. J. Rheol..

[28] Holly E.E., Venkataraman S.K., Chambon F., Winter H.H. (1988). Fourier Transform Mechanical Spectroscopy of Viscoelastic Materials with Transient Structure. J. Non-Newton. Fluid Mech..

[29] Winter H.H. (1987). Evolution of rheology during chemical gelation. Permanent and Transient Networks.

[30] Winter H.H., Mours M. (1997). Rheology of Polymers near Liquid-Solid Transitions. Adv. Polym. Sci..

[31] Winter H.H. (1987). Can the Gel Point of a Cross-Linking Polymer Be Detected by the G′–G″ Crossover?. Polym. Eng. Sci..

[32] Gough L.J., Smith I.T. (1960). A Gel Point Method for the Estimation of Overall Apparent Activation Energies of Polymerization. J. Appl. Polym. Sci..

[33] Ross-Murphy S.B. (1998). Reversible and Irreversible Biopolymer Gels—Structure and Mechanical Properties. Ber. Bunsenges. Phys. Chem..

[34] Nnyigide O.S., Hyun K. (2020). The Rheological Properties and Gelation Kinetics of Corn Starch/Bovine Serum Albumin Blend. Korea-Aust. Rheol. J..

[35] Dewar M.J.S., Pierini A.B. (1984). Mechanism of the Diels-Alder Reaction. Studies of the Addition of Maleic Anhydride to Furan and Methylfurans. J. Am. Chem. Soc..

[36] Liu Y.-L., Hsieh C.-Y., Chen Y.-W. (2006). Thermally Reversible Cross-Linked Polyamides and Thermo-Responsive Gels by Means of Diels–Alder Reaction. Polymer.

[37] Gandini A., Coelho D., Silvestre A.J.D. (2008). Reversible Click Chemistry at the Service of Macromolecular Materials. Part 1: Kinetics of the Diels–Alder Reaction Applied to Furan–Maleimide Model Compounds and Linear Polymerizations. Eur. Polym. J..

[38] Tian Q., Rong M.Z., Zhang M.Q., Yuan Y.C. (2010). Synthesis and Characterization of Epoxy with Improved Thermal Remendability Based on Diels-Alder Reaction. Polym. Int..

[39] Park J.S., Yun D.H., Ko T.W., Park Y.S., Woo J.W. (2013). Kinetic Study of the Diels-Alder Reaction of Cyclopentadiene with Bis(2-Ethylhexyl) Maleate. Adv. Mater. Res..

[40] Liu S., Liu X., He Z., Liu L., Niu H. (2020). Thermoreversible Cross-Linking of Ethylene/Propylene Copolymers Based on Diels–Alder Chemistry: The Cross-Linking Reaction Kinetics. Polym. Chem..

[41] Liu Y.-L., Hsieh C.-Y. (2006). Crosslinked Epoxy Materials Exhibiting Thermal Remendablility and Removability from Multifunctional Maleimide and Furan Compounds. J. Polym. Sci. Part A Polym. Chem..

[42] Engberts J.B.F.N. (1995). Diels-Alder Reactions in Water: Enforced Hydrophobic Interaction and Hydrogen Bonding. Pure Appl. Chem..

[43] Fringuelli F., Taticchi A. (2001). Diels–Alder Reaction in Unconventional Reaction Media. The Diels–Alder Reaction.

[44] Graziano G. (2004). Rate Enhancement of Diels–Alder Reactions in Aqueous Solutions. J. Phys. Org. Chem..

[45] Kwart H., Burchuk I. (1952). Isomerism and Adduct Stability in the Diels—Alder Reaction. 1a I. The Adducts of Furan and Maleimide. J. Am. Chem. Soc..

[46] Martin J.G., Hill R.K. (1961). Stereochemistry of the Diels-Alder Reaction. Chem. Rev..

[47] Rulíšek L., Šebek P., Havlas Z., Hrabal R., Čapek P., Svatoš A. (2005). An Experimental and Theoretical Study of Stereoselectivity of Furan–Maleic Anhydride and Furan–Maleimide Diels–Alder Reactions. J. Org. Chem..

[48] Cioc R.C., Lutz M., Pidko E.A., Crockatt M., van der Waal J.C., Bruijnincx P.C.A. (2021). Direct Diels–Alder Reactions of Furfural Derivatives with Maleimides. Green Chem..

[49] Buonerba A., Lapenta R., Ortega Sánchez S., Capacchione C., Milione S., Grassi A. (2017). A Comprehensive Depiction of the Furan-Maleimide Coupling via Kinetic and Thermodynamic Investigations of the Diels-Alder Reaction of Poly(Styrene–*co*-2-Vinylfuran) with Maleimides. ChemistrySelect.

[50] Cuvellier A., Verhelle R., Brancart J., Vanderborght B., Assche G.V., Rahier H. (2019). The Influence of Stereochemistry on the Reactivity of the Diels–Alder Cycloaddition and the Implications for Reversible Network Polymerization. Polym. Chem..

[51] Mangialetto J., Verhelle R., Van Assche G., Van den Brande N., Van Mele B. (2021). Time-Temperature-Transformation, Temperature-Conversion-Transformation, and Continuous-Heating-Transformation Diagrams of Reversible Covalent Polymer Networks. Macromolecules.

[52] Defize T., Thomassin J.-M., Alexandre M., Gilbert B., Riva R., Jérôme C. (2016). Comprehensive Study of the Thermo-Reversibility of Diels–Alder Based PCL Polymer Networks. Polymer.

[53] Guigo N., Sbirrazzuoli N., Vyazovkin S. (2012). Gelation on Heating of Supercooled Gelatin Solutions. Macromol. Rapid Commun..

[54] Chen K., Baker A.N., Vyazovkin S. (2009). Concentration Effect on Temperature Dependence of Gelation Rate in Aqueous Solutions of Methylcellulose. Macromol. Chem. Phys..

[55] Pescosolido L., Feruglio L., Farra R., Fiorentino S., Colombo I., Coviello T., Matricardi P., Hennink W.E., Vermonden T., Grassi M. (2012). Mesh Size Distribution Determination of Interpenetrating Polymer Network Hydrogels. Soft Matter.

[56] Kjøniksen A.-L., Nyström B. (1996). Effects of Polymer Concentration and Cross-Linking Density on Rheology of Chemically Cross-Linked Poly(Vinyl Alcohol) near the Gelation Threshold. Macromolecules.

[57] Elling B.R., Dichtel W.R. (2020). Reprocessable Cross-Linked Polymer Networks: Are Associative Exchange Mechanisms Desirable?. ACS Cent. Sci..

