# Supplementary material for: Chitosan Hydrogels Based on the Diels–Alder Click Reaction: Rheological and Kinetic Study
Source: Polymers (Basel). 2022 Mar 16;14(6):1202. doi: 10.3390/polym14061202 (PMC8955920; doi:10.3390/polym14061202)
Supplement: Supplementary file 1 [file polymers-14-01202-s001.zip › polymers-1591798-supplementary.pdf]

polymers

Article

Chitosan Hydrogels Based on the Diels–Alder Click Reaction: Rheological and Kinetic Study

Cinthya Ruiz-Pardo [1], Luisa Silva-Gutiérrez [1], Jaime Lizardi-Mendoza [1], Yolanda López-Franco [1], Carlos Peniche-Covas [2] and Waldo Argüelles-Monal [1,*]

[1] Grupo de Investigación en Biopolímeros, Centro de Investigación en Alimentación y Desarrollo A.C., Hermosillo 83304, Sonora, Mexico; cinthya.ruiz@estudiantes.ciad.mx (C.R.-P.); lsilva@ciad.mx (L.S.-G.); jalim@ciad.mx (J.L.-M.); lopezf@ciad.mx (Y.L.-F.); waldo@ciad.mx (W.A.-M.)
[2] Facultad de Química, Universidad de la Habana, Vedado 10400, La Habana, Cuba; cpeniche2015@yahoo.com
* Correspondence: waldo@ciad.mx

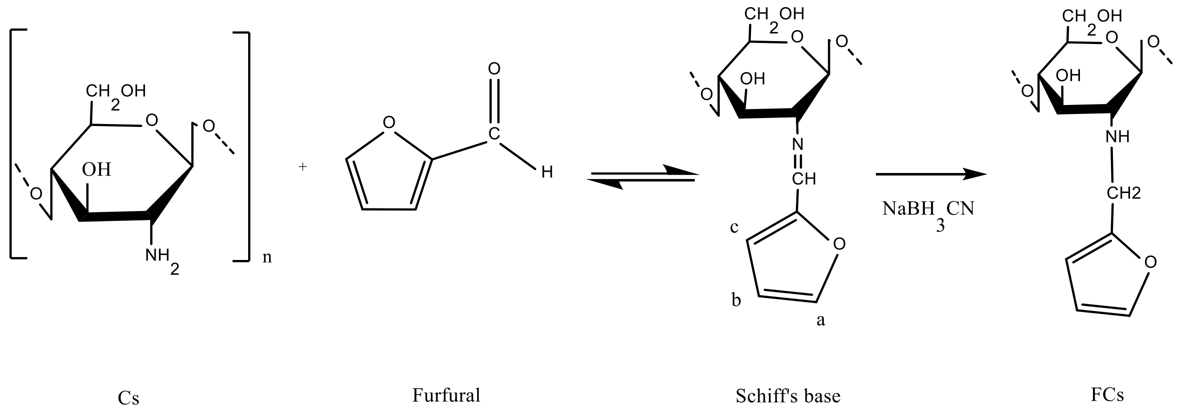

Scheme S 1. Synthesis of *N*-furfuryl chitosan.

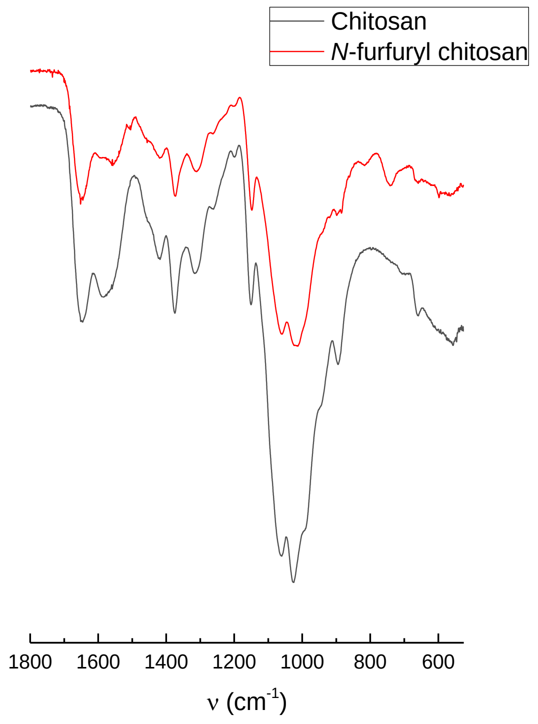

Figure S1. FT-IR spectra of chitosan and *N*-furfuryl chitosan.

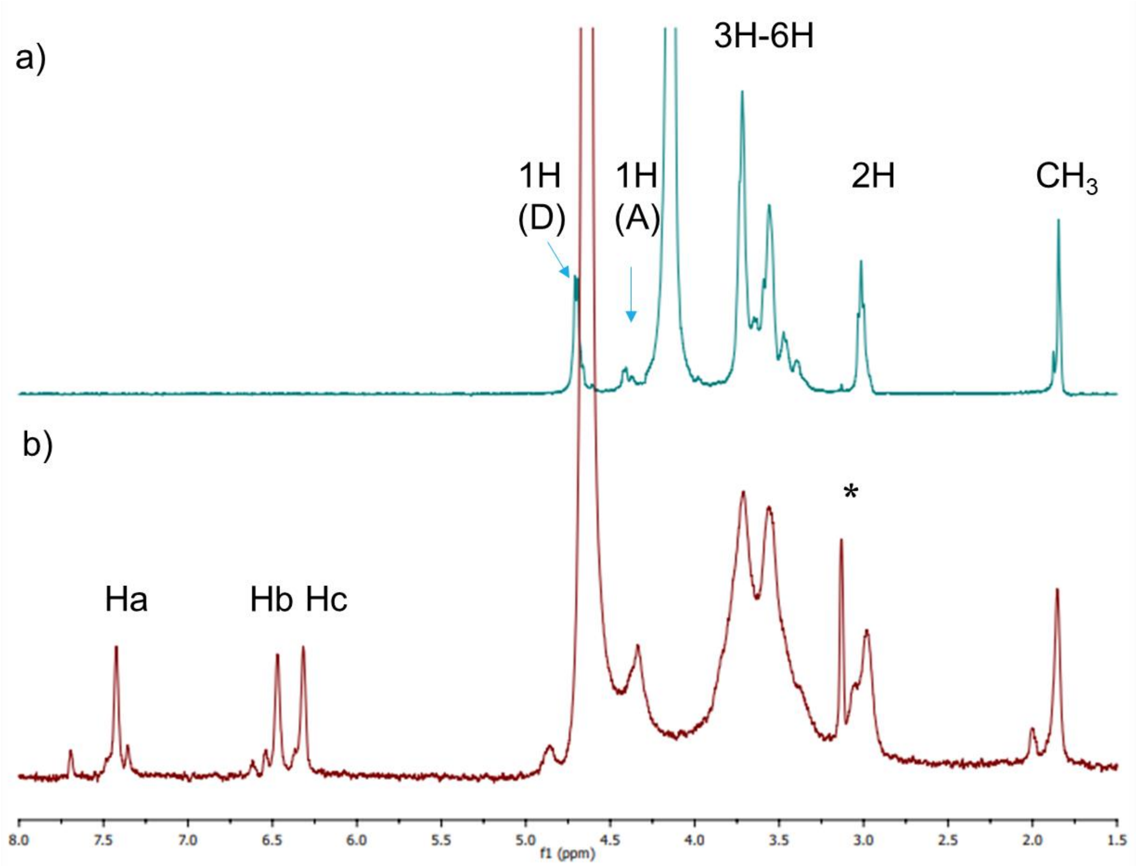

Figure S2. ^{1}H NMR spectra of (**a**) chitosan and (**b**) *N*-furfuryl chitosan.

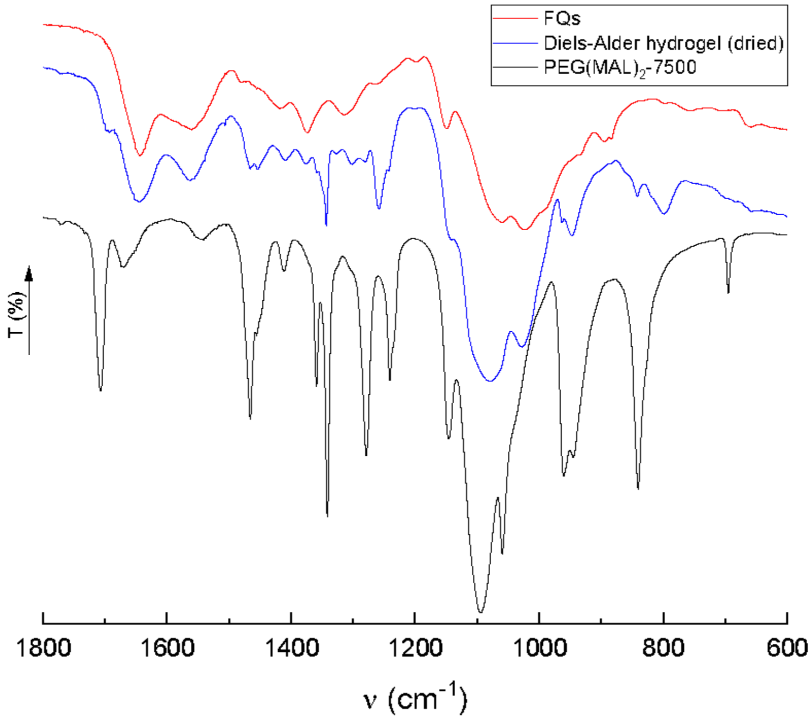

Figure S3. FTIR spectra of the *N*-furfuryl chitosan, PEG-bismaleimide and Diels–Alder cycloadduct.

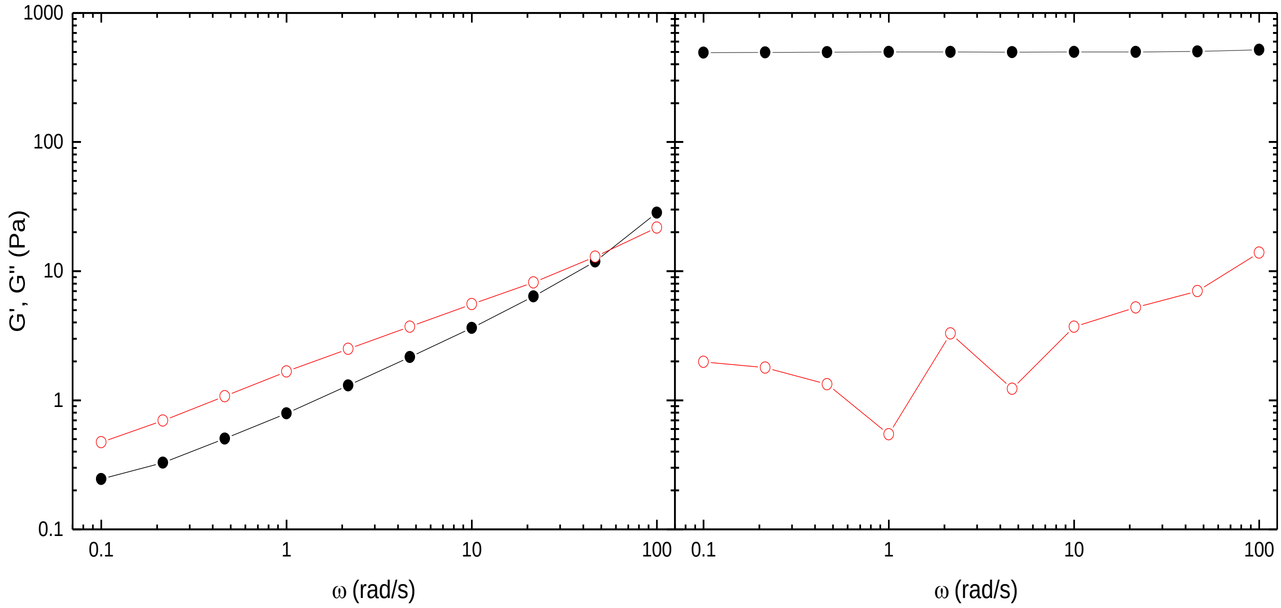

Figure S4. Mechanical spectra at 25 °C of the system of *N*-(furfuryl)-chitosan (1 %-*w*/*w*) and PEG(mal)2-7500 with R = 0.25 in 2% acetic acid a) before gelation and b) after gelation at 50 °C. G′, closed symbols, and G′′, open symbols.

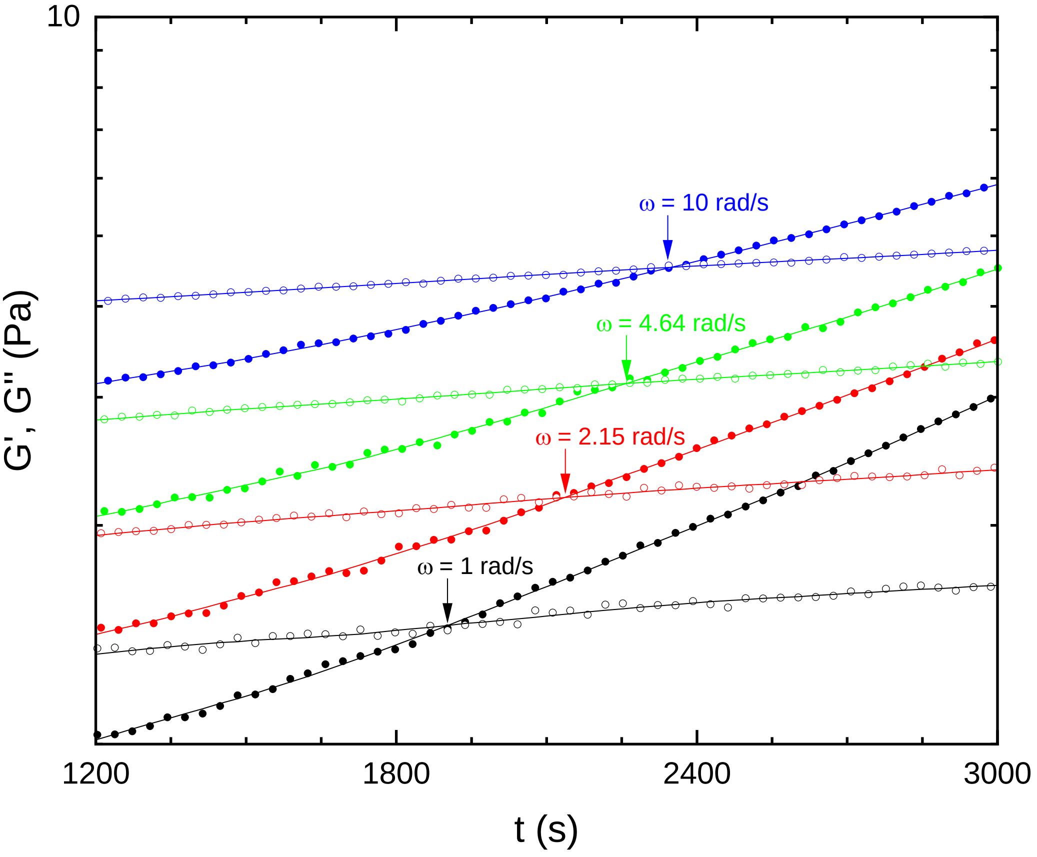

Figure S5. Storage and loss moduli as a function of the reaction time of the gelation process at 50 °C. *N*-furfuryl chitosan (21% degree of substitution) and PEG(mal) 2 -7500 dissolved in 2% acetic acid. Curves at different frequencies as marked in the graph. The frequency-dependence of moduli crossover point is obvious. Polymer concentration: 1 wt.%, R = 0.25.
